# MTEDS: Multivariant Time Series-Based Encoder-Decoder System for Anomaly Detection

**DOI:** 10.1155/2022/4728063

**Published:** 2022-09-28

**Authors:** A. Reyana, Sandeep Kautish, I. S. Yahia, Ali Wagdy Mohamed

**Affiliations:** ^1^Department of Computer Science and Engineering, Karunya Institute of Technology and Sciences, Coimbatore, Tamilnadu, India; ^2^Department of Computer Science and Engineering, LBEF Campus, Kathmandu, Nepal; ^3^Department of Physics, College of Science, King Khalid University, P.O. Box 9004, Abha 61413, Saudi Arabia; ^4^Research Center for Advanced Materials Science (RCAMS), King Khalid University, P.O. Box 9004, Abha 61413, Saudi Arabia; ^5^Department of Physics, Faculty of Education, Ain Shams University, Roxy, Cairo 11757, Egypt; ^6^Operations Research Department, Faculty of Graduate Studies for Statistical Research, Cairo University, Giza 12613, Egypt; ^7^Department of Mathematics and Actuarial Science School of Sciences Engineering, The American University in Cairo, Cairo 11835, Egypt

## Abstract

Intrusion detection systems examine the computer or network for potential security vulnerabilities. Time series data is real-valued. The nature of the data influences the type of anomaly detection. As a result, network anomalies are operations that deviate from the norm. These anomalies can cause a wide range of device malfunctions, overloads, and network intrusions. As a result of this, the network's normal operation and services will be disrupted. The paper proposes a new multi-variant time series-based encoder-decoder system for dealing with anomalies in time series data with multiple variables. As a result, to update network weights via backpropagation, a radical loss function is defined. Anomaly scores are used to evaluate performance. The anomaly score, according to the findings, is more stable and traceable, with fewer false positives and negatives. The proposed system's efficiency is compared to three existing approaches: Multiscaling Convolutional Recurrent Encoder-Decoder, Autoregressive Moving Average, and Long Short Term Medium-Encoder-Decoder. The results show that the proposed technique has the highest precision of 1 for a noise level of 0.2. Thus, it demonstrates greater precision for noise factors of 0.25, 0.3, 0.35, and 0.4, and its effectiveness.

## 1. Introduction

Anomaly detection is a widely researched topic in machine learning and is crucial in many areas such as fraud detection, cyber security, and complex system health monitoring. As a result, it continues to be an active and challenging research area. The industrial multivariate time series data is the focus of this paper. Industrial systems, such as power plants, wind turbines, and engines, generate large amounts of time series data during their normal operations thanks to an increasing number of sensors and cost-effective data transmission and storage solutions. It is critical to monitor these systems for abnormal behavior, which, if not detected early, can have serious reliability consequences. Such relationships will not adhere to the learned representation in an abnormal situation, resulting in deviations. Today, new business opportunities are established through the use of the Internet and corporate networks. Numerous businesses and governments have established complex networks [1]. These networks are equipped with techniques for dispersed data storage, encryption, decryption, and authentication. Corporate networks are more accessible and utilize virtual private networks to enable employees to connect [2], thereby increasing the vulnerability of today's network to attackers. Network-based attacks are increasing in frequency and severity, resulting in increased financial losses. Firewalls have ceased to provide appropriate protection [3]. According to a recent survey, forty new vulnerabilities are uncovered each month. This unsecured environment has necessitated the development of intrusion detection and prevention systems.

Intrusion detection systems do an analysis of the computer or network for potential security vulnerabilities. It identifies actions that jeopardize the integrity, reliability, and confidentiality of the system [2]. Time-series data are real-valued. Time series data are frequently used in financial, medicinal, and meteorological applications [4]. Routers and switches are used to facilitate network communication. Each of these entities contributes to the network's behavior. Due to the dynamic nature of the Internet protocol, it is difficult to comprehend the system. To comprehend network behavior, it is vital to understand how network probes are used [5]. This is a characteristic of uncontrolled networks. With an understanding of the network's layout, it is simple to identify network irregularities and performance bottlenecks. The type of anomaly detection varies according to the nature of the data [5]. Thus, network anomalies are operations that depart from the usual. These abnormalities can result in a variety of device malfunctions, overloads, and network incursions, among other things. This will cause a disruption in the network's normal operation and services. The normal behavior of a network is determined by a variety of factors, including the volume of traffic, the type of application, and the type of network data [5].

Today's commercially available network management systems monitor network activity in real-time and alert users to irregularities. The network manager monitors the environment and generates alarms when an unusual one occurs. Anomalies in networks are defined by changes in the measured data. Anomalies in networks are classified into two categories [5]. The first category results in network failures and performance problems such as file server errors and congestion. This abnormality will affect all users who share the network's available capacity. The second type results in security difficulties such as denial of service assaults, intrusions, and so forth. The correct type of network information is critical for any anomaly detection system. The more precise the traffic behavior, the more accurate the detection of anomalies.

### 1.1. Our Contribution

- To develop a novel Multi-variant Time-series- based Encoder-Decoder System (MTEDS) for detecting anomalies.
- To construct multi-scale signature matrices to characterize multiple levels of the system statuses in different time steps.
- To define a radical loss function to update the network weights through backpropagation.
- To propose a technique that is noise-resistant and delivers a variety of anomaly scores based on the severity of the incident.
- To evaluate the performance of the proposed system in terms of anomaly score, noise factor, attention weight, anomaly count, precision, and recall. Finally, validate the proposed system against three existing approaches: Multiscaling Convolutional Recurrent Encoder-Decoder (MSCRED), Autoregressive Moving Average (ARMA), and Long Short Term Medium-Encoder-Decoder.

The remainder of the article's section is organized as follows: Section 2 discusses the various studies on time series-based anomaly detection that have been addressed. The proposed research methodology is illustrated in Section 3, followed by the results and discussion in Section 4, and finally the conclusion in Section 5.

## 2. Related Works

Anomaly detection in time series data has attracted the interest of a growing number of researchers in recent years. Numerous accessible techniques have difficulty in resolving these issues. Wang et al. in [6] created an online self-learning anomaly detection method to detect the abnormality automatically. Time series data are generated as a result of a succession of events, equipment sensors, or other sources. Anomalies can be structural, anomalous, or point in nature. In this case, the data points diverge. Structural does not follow the expected pattern, and anomalous time series contain deviations. In real-world applications, the difficulty is the occurrence of new anomalies with unknown behavior and duration. The study proposes techniques for detecting anomalies in univariate and multivariate time series. The algorithm is capable of detecting time series, locations, and sensors associated with them. Two algorithms were developed: SLADE TS and MTS. Initially, the algorithm splits the time series automatically into variable-length windows. Objects' behavioral patterns are not identified in all dimensions. However, accuracy and the determination of precise measurements and locations continue to be a challenge.

Memarzadeh et al. in [7] concentrated on identifying the National Airspace System's vulnerabilities. It is difficult to detect an anomaly in a high-dimensional heterogeneous time series. To capture the complicated patterns, the authors trained a convolutional variational auto-encoder. The model is composed of two components: an encoder that converts the original data space to a low-dimensional latent space, and a decoder that reconstructs the original data. The model optimizes mapping and quantifies the reconstruction error. This method is used to detect anomalies. Although the model performs well with high-dimensional data, dealing with heterogeneous multivariate time series data remains a barrier to vulnerability discovery. In [8] the authors surveyed the most advanced anomaly detection algorithms in the aviation domains. Anomaly detection is critical in a variety of industries. Due to the complexity of aviation's traffic circumstances, current anomaly detection approaches are insufficient. They evaluated current improvements in deep learning algorithms for scaling high-dimensional data in light of the availability of a vast amount of sensor data. Thus, it is clear that detecting anomalies in real-world applications continue to be a challenge.

Anomaly detection is the process of identifying anomalous behavior within a specified period. Li et al. in [9] proposed a method for detecting anomalies in multivariate time series using clustering. Due to the temporal link between points and individual time series, multivariate time series detection remains difficult. The authors classified aberrant signals into two categories based on their amplitude and shape. The fixed-length sliding window is initially constructed from multivariate subsequences. Subsequently, expanded Fuzzy-C-Means clustering is used. Thus, the anomaly score is identified, which quantifies the deviation from normal data. To deal with the initial feature space, the clustering procedure employs the Euclidean distance. Correlation of time series encapsulates shape information and identifies time series-based dissimilarity. Based on simulated datasets, the technique was tested. A confidence index was used to determine the cluster numbers and the interval length. As a result, the proposed approach recognized shape irregularities. While the approach performs admirably, it is limited in that it does not reveal the dimension of multivariate time series. Additionally, heavy computation generates overhead and is a time-consuming operation.

Khan et al. in [10] concentrated on anomaly detection in the aerospace domain, with a particular emphasis on machine learning techniques. Unsupervised anomaly detection is critical for detecting unknown cases in multivariate time series. The authors explored a variety of issues in their attempt to make a credible prognosis of the scenario. These include data availability, unsupervised learning for processing vast amounts of heterogeneous data, measurement error, and the complexity of learning real-time patterns. Despite the algorithms' strong results, determining the appropriate threshold level for isolating the anomaly remains a challenge. In [11] Han proposed an unsupervised method for detecting medical anomalies through the use of generative adversarial networks. The innovative method consists of two steps and is capable of detecting a variety of ailments. This phase covers the rebuilding of unhealthy and aberrant scans. The diagnosis phase then establishes the ground truth. The receiver operating characteristic (ROC) and area under the curve (AUC) were calculated. The study made use of the OASIS 3 clinical dementia rating dataset. Thus, the disease was diagnosed using many MRI sequence images. In [12] Fan and others examined the autoencoder's performance in detecting anomalies and constructing energy data. Autoencoders are a kind of neural network that is capable of doing unsupervised learning. There is no label variable in the learning process. The encoder takes identical inputs and converts them to high-level characteristics. The auto encoders deal with a variety of issues and are composed of fully connected layers. The autoencoder ensemble approach analyses energy data for abnormalities. The system was able to detect anomalies related to abnormal occurrences, operational errors, and inefficiency in the control strategy, among other things. However, developing unsupervised anomaly detection autoencoders remains a difficulty. The authors [13] concentrated on developing a secure and trustworthy system through the use of an anomaly detection technique. They deployed a DeepLog, a deep neural network with Long Short Term Memory that was used to represent the system log. DeepLog automatically discovers log patterns based on the log data. The approach makes use of the massive amount of system logs. The reason for this is that log data is unstructured and varies by system. It's difficult to diagnose unstructured log files. The recurrent neural network loops back from the previous state to the present input and maintains a record of previous predictions. The long-term repercussions are recalled. In a streaming approach, DeepLog trains on small data sets and recognizes the standard log sequence. However, the scientists noted that employing a recurrent neural network to diagnose a system remained challenging.

Al Osman and other experts in [14] place a premium on stress management through the use of ubiquitous biofeedback serious games. The device continuously monitors the subject's mental stress by monitoring their heart rate, respiration rate, and degree of activity. As with a biological sensory paradigm, the approach operates as a loop between the body and the brain. It takes physiological data throughout game sessions. Mental stress is monitored using electrodermal sensors. The two-way communication between the game and the player is established. As the player becomes immersed in the game, biological signals are captured and the time required to assess relaxation is recorded. The u-biofeedback system achieves continuous monitoring ubiquity. The algorithm has two modes of operation: limited and indefinite. The limited play can establish their monitoring period for a short time, whereas with indefinite, monitoring continues for the duration of the game. As a result, the technique aids individuals in determining their stress levels. With the proliferation of interconnected devices and sensors in the industrial system, it is critical to monitor for threats. This is especially crucial in power grids, water treatment facilities, and communication networks because as complexity develops, it becomes more difficult to spot anomalies. In [15], they introduced a novel graph deviation network to learn the sensor relationship graph. Four components comprise the approach: sensor embedding, graph structure learning, attention-based forecasting, and deviation score. The trials used datasets from water treatment plants to demonstrate the accuracy of the anomaly detection algorithm. While the model comprehends the anomaly, the authors' issue about improving anomaly detection with hyperparameters remains unresolved. The authors in [16] introduced a novel GAN model for anomaly detection and a time series imaging localization framework. The sequence of distance images from one to the next is memorized. The encoder and decoder convert multivariate time series to two-dimensional pictures. The encoder's pointwise convolution ensures that temporal information is encoded. The experiment is conducted using the smart power plant dataset. As a result, the generator generates a sequence of distance images. However, detecting anomalies in image localization continues to be a challenge.

Zhang et al. [17] noted that multivariate time series data are becoming more prevalent in the actual world. For instance, power plants and wearable technology. When dealing with multivariate time series, the anomaly is identified, and the problem is capturing the temporal dependency inherent in each time series. Additionally, the system should be noise-resistant. Although the number of unsupervised anomaly detection algorithms continues to grow. The authors proposed the use of a Multiscale Convolutional Recurrent Encoder-Decoder (MSCRED) for anomaly detection in multivariate time series data. To begin, the technique generates a signature matrix that is used to characterize multi-level system states. Following that, the time-series correlation is encoded using the signature matrices in a convolutional encoder. Precision, recall, and *F*1 scores were determined during the examination. Thus, when applied to synthetic datasets, the model outperformed state-of-the-art approaches. In [18] the authors suggested a one-step-ahead load forecasting algorithm for estimating electricity requirements. The framework incorporates both the linear autoregressive and artificial neural network techniques. The Bayesian information criterion is used to counteract human-induced random events. The experimental results showed that the study is effective at selecting features from nonstationary time series data. Utilizing sophisticated approaches for anomaly prediction in the future remains a challenge. The Temporal Hierarchical One-Class (THOC) network was proposed in [19]. Initially, it used a recurrent neural network to extract multi-scale temporal properties from time series. The approach performed hierarchical clustering using the intermediate layer characteristics. This is because the upper layers include the characteristics with the lowest resolution. Multiple hyperspheres define the normal behaviors for each resolution. The intricate properties of real-world systems are captured through the use of multiscale support vector data descriptions. The one-class objectives are stated, and the end-to-end training of multiscale features and hypersphere centers is permitted. The deviation score is used to detect anomalies, and the approach outperforms contemporary state-of-the-art approaches. In [20] developed an improved deep model for detecting time series anomalies. The method was applied to a collection of hyperspheres with a hierarchical structure. To have access to all of time's events. The representation of features was enhanced to account for orthogonality and temporal self-supervision loss. Each component's effectiveness was evaluated.

Li et al. in [21] offered two solutions to the challenge of segmenting moving objects. The authors began with a transposed convolutional neural network and then added a Features Pooling Module. The first stage involved mapping the features to a pixel-level foreground probability map using the multiscale feature encoder and decoder. Then the binary segmentation labels were created. The proposed approaches are straightforward and operate with networks with numerous inputs. The review was conducted using publicly available datasets from CDnet2014, SBI2015, and UCSD. In [22] discussed the difficulties associated with anomaly detection in the Internet of Things. The Internet of Things facilitates data communication without the direct involvement of human agents. The gadgets interact with the environment via sensors, actuators, computers, and smart objects. The authors have highlighted several significant obstacles, including (i) Real-time processing, i.e., anomaly detection, which requires real-time decision-making. When the detector takes an extended period, the system fails. (ii) Incremental technique, i.e., maintaining the complete dataset for analysis is challenging, and using an incremental approach reduces memory needs. (iii) Online adaptive learning necessitates system reconfiguration.

Multivariate data on the environment and other sensor information must be addressed [23]. Thus, the author's point on historical anomaly detection for univariate exists. Yet multivariate data stream-based anomaly detection continues to be a difficult problem, which motivated this research. Although intrusion detection systems have evolved, next-generation anomaly detection systems face numerous obstacles. These include legacy intrusion detection systems that are incapable of adapting to emerging network paradigms such as wireless and mobile networks. They have not been scaled to meet the requirements of high-speed networks. Noise, constantly shifting traffic patterns, and massive networks go unaddressed [2]. False alarms must be suppressed. Despite the multiple methodologies used, there are currently no universally accepted criteria for evaluating intrusion detection systems. As a result, evaluating an intrusion detection system fairly using systematic criteria continues to be a challenge. Additionally, in today's actual network environment, having the necessary data for IDS evaluation is crucial [24]. Apart from this, another significant obstacle is the proliferation of attacks in various forms. The majority of intrusion detection systems are ineffective at detecting intrusions on time. Thus, the most difficult problem is multivariant time-series detection. While considerable work has been done in this area, additional research is necessary to discover realistically effective solutions [25].

## 3. Materials and Methods

Today's real-world systems generate an increasing amount of data. Identifying anomalous states and isolating the underlying reasons is challenging. The purpose of this study is to detect anomalies in multivariate time series data using a synthetic dataset of real power plants. In modern industrial facilities, complex systems are used. Numerous sensors are used to monitor the activity of these systems. The sensors generate multivariate time series data, which must be analyzed for anomalies [26]. In today's industrial world, multivariate time series data are generated by monitoring the behavior of complex systems, for instance, temperature and pressure sensor readings in a power plant. These systems must be repaired immediately to address the difficulties mentioned. Managing this procedure and undertaking exact detection would come at a significant financial and human cost [23]. In the long run, small perturbations in a system cannot be viewed as system failures. As a result, several different anomaly scores must be offered to aid system operations. This enables operators to take remedial action and rectify any difficulties that may arise. The discovered anomaly score shall indicate the system failure based on the sensor data. Accurate and prompt judgments are required to avert catastrophic losses in the case of a power plant. Additionally, it is required to locate and repair the sensors responsible for the anomaly. Anomaly detection at various severity levels will finally reveal the robustness of the systems [27]. Systems that self-detect abnormalities are constructed using supervised algorithms. Although unsupervised algorithms exist, they face significant difficulties. In the event of an abnormality, multivariate time series data is critical for determining the aberrant condition and establishing the core causes [28, 29]. Constructing time series-based systems is challenging because it requires the intercorrelation of numerous time-series data. Thus, to achieve the abnormal status of identifying and diagnosing anomalies based on time series, this research study proposes a Multi-variant Time series-based Encoder-Decoder System (MTEDS), in which the system utilizes time-series inter-correlation with the help of the state vector and signature matrices, which aids in analyzing the anomaly score based on the client matrix series variation (residual matrices) in our dataset. Following an adequate number of training sessions, the network parameters are used to infer the reconstructed signature matrices for the validation and test data. Then, using latent vector characteristics, the anticipated number of anomalies may be checked against the enormous variety of Anomalies identified using this reconstruction [6].

**Figure 1:**
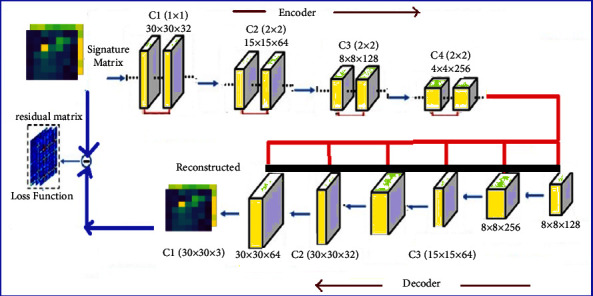
Framework of the proposed MTED.

The work introduces a novel MTEDS for handling anomalies in time series data with multiple variables. It begins by constructing multi-scale signature matrices to detect anomalies in the system's condition in a variety of time series. This value represents the degree to which the system is anomalous. Following that, the variational autoencoder is used to decode the time series data's correlation patterns. A convolutional long-short-term memory is used to capture the temporal patterns. Thus, the correlation between sensors and temporal information is sent to the Variational auto decoder for the reconstruction of the signature and residual matrices. The signature matrices are labeled S_*normal*_ and S_*malicious*_, indicating which actions are considered normal and deleterious. Thus, the MTEDS was designed for anomaly detection and problem diagnosis. To evaluate the proposed MaVES system's performance, it is compared to three existing approaches: Multiscaling Convolutional Recurrent Encoder-Decoder (MSCRED), Autoregressive Moving Average (ARMA), and Long Short Term Medium-Encoder-Decoder (LSTM-ED). The proposed MTEDS framework is represented in Figure 1.

Initially, the system generates multi-scale signature matrices. The input is fed into the encoding vector, and each dimension represents a learned attribute. For each dimension, the suggested encoder employs a single value. Similarly, the decoder recreates the input's original value. The variation autoencoder depicts the observation in latent space, with the properties of the observation being used to calculate the probability distribution. Normally, the latent state for the input vector creation is chosen at random during the decoding phase. It is the feasible range of values as output feed into the decoder in the proposed model. Each time series' temporal dependency is correlated, and the system is noise-resistant. The various components of the proposed MTEDS framework are herewith described below.

### 3.1. Signature Matrices

The system status is characterized using different time series. Here for a multivariate time series of *m* − *w* to *m*, we construct *n* × *n* signature matrices *S*^*m*^. For a two-give time series as mentioned in

(1) $$\begin{array}{c} p_{i}^{w}=\left(p_{i}^{m-w},p_{i}^{m-w-1},\cdots p_{i}^{w}\right)\text{,} \end{array}$$

(2) $$\begin{array}{c} q_{j}^{w}=\left(q_{j}^{m-w},q_{j}^{m-w-1},\cdots q_{j}^{w}\right)\text{.} \end{array}$$

The correlation *S*_*ij*_^*m*^ € *S*^*m*^ is calculated using

(3) $$\begin{array}{c} s_{ij}^{m}=\frac{\overset{w}{\underset{\delta =0}{\sum }}p_{i}^{m-\delta }p_{j}^{m-\delta }}{k}\text{.} \end{array}$$

Here the rescaling factor is *k* and *k*=*w*. The signature matrix is assumed to be robust to noise and it does not capture the shape similarities. The segment interval is assumed as 10 and the signature matrix for *s* = 3 is constructed for lengths *w*=10, 30, and 60.

### 3.2. Variational Auto Encoder

The variational autoencoder is a latent model that uses the regularized version of the autoencoder. The *z*, hidden codes enforce to draw samples using regular sampling. Here *φ*(*p*) is the deterministic function, and the recognition model *t*(*z*/*p*). The model employs a Gaussian disturbance over *z* with a neural network condition on *p*. The calculation is expressed in [21].

(4) $$\begin{array}{c} V\left(\theta ,p\right)=-KV\left(b_{\theta }\left(\frac{z}{p}\right)a\left(z\right)\right)+Eb_{\theta }\left(\frac{z}{p}\right)\left[{loga}_{\theta }\left(\frac{p}{z}\right)\right]\leq loga\left(p\right)\text{.} \end{array}$$

This decodes every point in the latent space and has a reasonable probability.

### 3.3. Convolutional LSTM

The convolutional LSTM adapts hidden states across various time series. The feature maps *p*^*m*,*l*^ from the lth layer of the convolutional neural network and *H*^*m*−1,*l*^ the hidden state belongs to *R*^*n*1×*n*1×*d*1^. Therefore, the calculations are expressed in

(5) $$\begin{array}{c} \overset{\wedge }{H^{m,l}}=\underset{i\in \left(m-h,m\right)}{\sum }\alpha^{i},H^{i,l}\text{,} \end{array}$$

where *α*^*i*^ the previous weight is expressed in

(6) $$\begin{array}{c} \alpha^{i}=\frac{\exp\;\left\{vec\left(H^{m,l}\right)T\;vec\left(H^{i,l}\right)/k\right\}}{\underset{i\in \left(m-h,m\right)}{\sum }\exp\;\left\{vec\left(H^{m,l}\right)T\;vec\left(H^{i,l}\right)/k\right\}}\text{,} \end{array}$$

where *vec*() denotes the vector, *k* the rescaling factor. Thus the spatial patterns of the signature matrix are mapped with the temporal information at the convolutional layer.

### 3.4. Loss Function

The loss function defines the reconstruction errors over the signature matrix and is expressed in

(7) $$\begin{array}{c} L_{f}=\sum_{m}\overset{s}{\underset{c=1}{\sum }}{\left\|p_{:;:c}^{m,0}-{\overset{\wedge }{p}}_{:;:c}^{m,0}\right\|}_{F}^{2}\text{,} \end{array}$$

where *c* € *R*^*n×n*^. The Adam optimization minimizes the loss function by employing stochastic gradient descent. The number of training epochs is sufficiently obtained the signature matrix is validated using the learned neural network. Based on the obtained residual matrix the anomaly is detected. Further, the performance of the proposed technique was evaluated using metrics such as anomaly score, noise factor, attention weight, anomaly count, precision, and recall.

## 4. Results and Discussion

This section describes the experimental setup, datasets, and various evaluation metrics.

### 4.1. Datasets

The synthetic dataset of a power plant was used. For the dataset the time series was formulated as follows:

  For *S*_rand_=0; *S*(*m*)={sin [*m* − *m*_0_/*w*]+*λ* · *ϵ*}
  For *S*_rand_=1; *S*(*m*)={cos [*m* − *m*_0_/*w*]+*λ* · *ϵ*}

The *S*(*m*) value captures three sets of multivariate time series attributes. *C*1 is the temporal pattern simulation using trigonometric function; *C*2 is the time delay of the periodic cycles i.e. *m*_0_*ϵ* [50, 100] and *w* *ϵ* [40, 50]; *C*3 is the random Gaussian noise *n*(0, 1) and the scaling factor *λ* is set as 0.3. The random selection of pairs of frequency and time at certain times leads to low and high correlations. Thus, the synthetic dataset considered consists of 30-time series, 20000 points, and 4 anomalies.

### 4.2. Experimental Setup

The experiment was conducted on a PC with a 3.6 GHz CPU, 12 GB of RAM, and Windows 10 OS. All codes are implemented in python language respectively.

### 4.3. Performance Evaluation Criteria and Parameters

**Figure 2:**
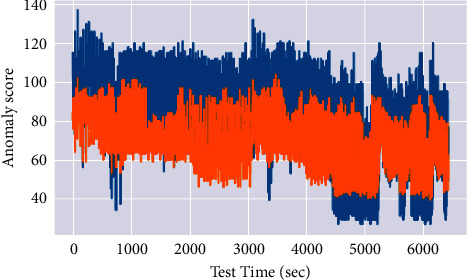
Anomaly score 1 results.

**Figure 3:**
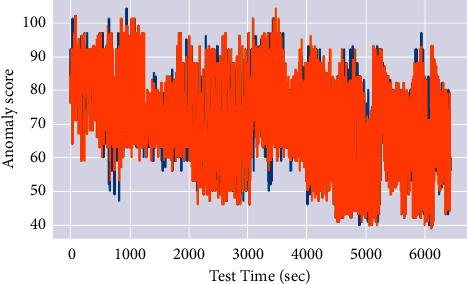
Anomaly score 2 results.

The experiments were conducted to evaluate the various performance metrics such as anomaly score, noise factor, attention weight, anomaly count, precision, and recall. Finally, validate the proposed system against three existing approaches: Multiscaling Convolutional Recurrent Encoder-Decoder (MSCRED), Autoregressive Moving Average (ARMA), and Long Short Term Medium-Encoder-Decoder. Figures 2 and 3 depict the anomaly score results of the considered synthetic dataset.

Multiple levels of system status in various periods must be characterized. Following that, given the signature matrices, a convolutional encoder is used to encode the inter-time-series correlations based on the Client metric and changes, resulting in an alert and an increase in the anomaly score. The anomaly scores are used to assess the proposed MTEDS performance. The results in Figures 2 and 3 show that the suggested model is effective in terms of inter-sensor correlation and temporal patterns. The results reveal that the anomaly score is more consistent and detectable, with fewer false positives and negatives.

**Figure 4:**
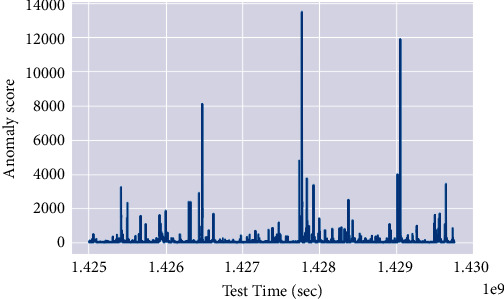
Mapping of anomaly detected against the threshold.

The anomaly periods are shown in blue in Figure 4, and the cutting threshold is shown in white. The results demonstrate the efficacy of the proposed model.

**Figure 5:**
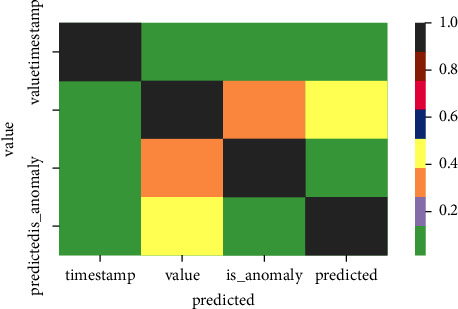
Generated matrix.

The matrix generated is shown in Figure 5 and Table 1. The matrices obtained from the residual and signature time series are validated based on the large variety of anomalies detected. Based on this, the reconstruction takes place, which is a latent vector where the predicted amount of the variation series is increased, and the reconstruction is enabled with the next set of matrices. The network weights are updated using backpropagation after defining a differentiable loss function. Further variational autoencoders reduce the reconstruction error between the network's input and output by minimizing the generative model parameters *m* simultaneously. With the use of residual matrices, the evaluation of the vector time series sequence is subdivided into several parts with the help of the residual matrices by this the differentiation of how the anomaly variates are determined.

**Figure 6:**
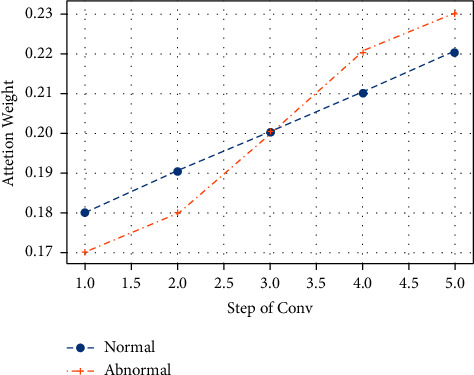
Distribution of attention weights.

The distribution of attention weights over the previous timestamps is shown in Table 2 and Figure 6. Both the normal and abnormal attention weights are displayed separately. Even minor changes in the system status cause the attention to become more sensitive, which is useful for anomaly detection. The fourth and fifth-time steps, for instance, have an abnormal status of 0.22 and 0.23, respectively, whereas the first and second-time steps appear to be normal, and therefore the weights are assigned less than the normal segments.

**Figure 7:**
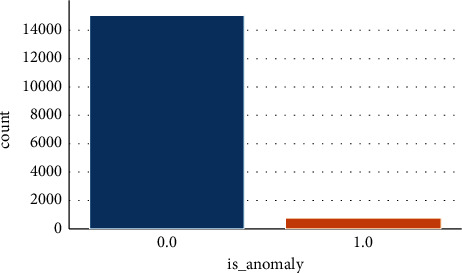
Performance evaluation of proposed MTEDS.

Table 3 and Figure 7 illustrate the performance results of the proposed MTEDS in terms of noise factors, precision, and is anomaly. The noise factor found appears to be relatively low, ranging between 0.2 and 0.45. The high precision value of 1 can be found in the majority of timestamps. As a result, the proposed system's efficiency is demonstrated. Further, the efficiency of the proposed MTEDS is compared with the three existing approaches: Multiscaling Convolutional Recurrent Encoder-Decoder (MSCRED), Autoregressive Moving Average (ARMA), and Long Short Term Medium-Encoder-Decoder.

**Figure 8:**
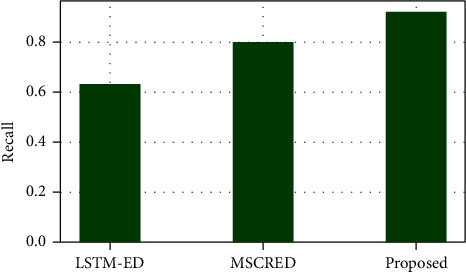
Recall measure comparison of the proposed MTEDS with the existing one.

Figure 8 shows the recall measure in comparison to the two previous systems, LSTM-ED and MSCRED. When compared to the MSCRED and LSTM-ED, which have recall measures of 0.81 and 0.63, respectively, the proposed MTEDS has the highest recall measure of 0.81. As a result, the proposed system's efficiency is demonstrated.

**Figure 9:**
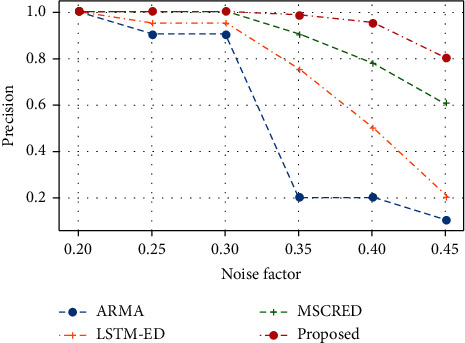
Precision measure comparison of the proposed MaVES with the existing.

The precision measure comparison of the proposed MTEDS with the existing system is presented in Table 4 and Figure 9. The proposed approach had a precision of 1 for a noise level of 0.2. The precision value decreases as the noise level rises. However, for noise factors of 0.25, 0.3, 0.35, and 0.4, the proposed system had greater precision of 1, 1, 0.98, and 0.95, respectively. When compared to the proposed, the MSCRED has a precision of 1, 0.90, and 0.378. The least efficient systems were LSTM-ED and ARMA, demonstrating the efficacy of the proposed system.

## 5. Conclusion

To detect anomalies in multivariate time series data, the proposed research study MTEDS was implemented. It creates multi-scale signature matrices to describe many levels of system status in various time steps. As a result, a radical loss function is defined to update network weights via backpropagation. Multiple levels of system status were assessed over time. The suggested MTEDS performance is evaluated using anomaly scores. According to the findings, the anomaly score is more stable and traceable, with fewer false positives and negatives. The proposed technique provides the highest precision of 1 for a noise level of 0.2, according to the results. As the noise level increases, the precision value lowers. The proposed system, however, showed greater precision for noise factors of 0.25, 0.3, 0.35, and 0.4, proving its effectiveness. Although the proposed system performed better in terms of anomaly detection, the notion of anomaly would change as the dataset changes. It could be extended in the future to improve the method by applying it to different real-time datasets. Scaling and deploying the algorithm in complex operations might also be addressed while diagnosing the core cause of the problem. Other variables that could be examined for performance evaluation include latency and power analysis.

## Figures and Tables

**Table 1:** Generated matrix.

*Time stamp*	1	0.1	0.1	0.1
*Value*	0.1	1	0.3	0.4
*Anomaly*	0.1	3	1	0.1
*Predict*	0.1	0.4	0.1	1
	*Timestamp*	*Value*	*is anomaly*	*Predicted*

**Table 2:** Distribution of attention weights.

Step of conv	Attention weight	
	Normal	Abnormal
	0.18	0.17
	0.19	0.18
	0.2	0.2
	0.21	0.22
	0.22	0.23

**Table 3:** Performance evaluation of MTEDS.

Proposed MTEDS	
Noise factor	Precision
0.2	**1**
0.25	**1**
0.3	**1**
0.35	**0.98**
0.4	**0.95**
0.45	**0.8**

**Table 4:** Precision measure comparison of the proposed with the existing system.

	Precision			
Noise factor	ARMA	LSTM-ED	MSCRED	*Proposed*
0.2	1	1	1	**1**
0.25	0.9	0.95	1	**1**
0.3	0.9	0.95	1	**1**
0.35	0.2	0.75	0.9	**0.98**
0.4	0.2	0.5	0.78	**0.95**
0.45	0.1	0.2	0.6	**0.8**

## Data Availability

The data used to support the findings of this study are available on request.
